# Supplementary material for: INGAP-Peptide Variants as a Novel Therapy for Type 1 Diabetes: Effect on Human Islet Insulin Secretion and Gene Expression
Source: Pharmaceutics. 2022 Aug 31;14(9):1833. doi: 10.3390/pharmaceutics14091833 (PMC9502412; doi:10.3390/pharmaceutics14091833)
Supplement: Supplementary file 1 [file pharmaceutics-14-01833-s001.zip › pharmaceutics-1852807-supplementary.pdf]

Supporting Information

INGAP-Peptide Variants as a Novel Therapy for Type 1 Diabetes: Effect on Human Islet Insulin Secretion and Gene Expression

James M. Porter[a], *Léa Guerassimoff*[b,d], *Francisco Rafael Castiello*[a], *André Charette*[d] *and Maryam Tabrizian*[a,b]*

a) Dept. of Biological and Biomedical Engineering, Faculty of Medicine and Health Sciences, McGill University, 740 Dr. Penfield, Montreal, Quebec, H3A 0G4, Canada

b) Faculty of Dental Medicine and Oral Health Sciences, McGill University, 001 McGill College Ave, Montreal, Quebec, H3A 1G1

d) l'Université de Montréal, Campus MIL, 1375 Ave. Thérèse-Lavoie-Roux, Montréal, QC H2V 0B3

* Corresponding Author: maryam.tabrizian@mcgill.ca

Sample Mass Spectrometry Data: I6 exact mass isomer

Exact Mass Report – I6

**Data File**	I6-1.d	**Sample Name**	I6-1
**Sample Type**	Sample	**Position**	P1-B1
**Analysis Date**	7/11/2019 9:50:04 AM	**User Name**	KG
**Acq Method**	ESI_POS_DI.m	**InstrumentName**	TOF 6224
**Comment**	MPA:H20+0.1%FA MPB:MeOH		

MS Spectrum

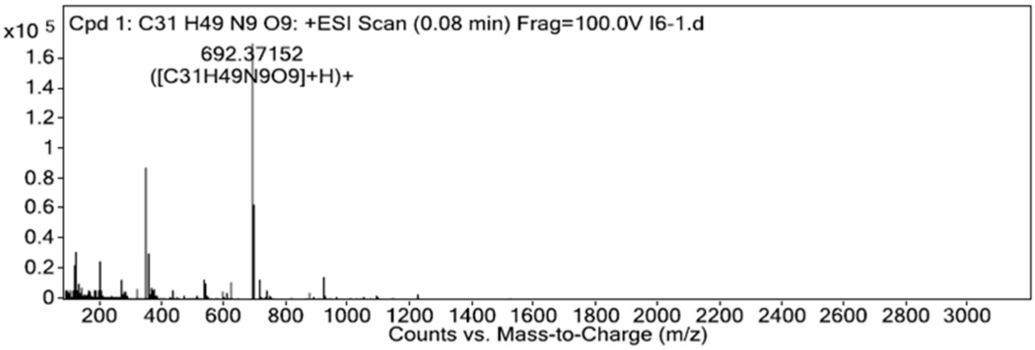

MS Zoomed Spectrum

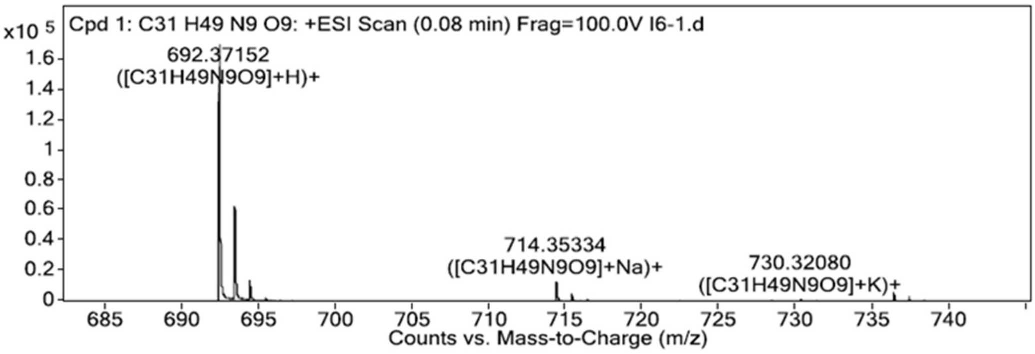

MS Spectrum Peak List

Ion	Formula	Abund	*Expe. m/z*	*Calc. m/z*	Diff(ppm)
(M+H)+	C31H49N9O9	171227.52	692.37152	692.3726	1.56
(M+Na)+	C31H49N9O9	13114.12	714.35334	714.35455	1.69

<u>Sample Purity Analysis</u>: I19 (after purification)

Purity

**Data File**	I19	**Sample Name**	I19
**Folder**	2019I_sep	**User Name**	LOUIZA
**Acq Method**	LC_ISO23_20min_col2	**DA Method**	
**Instrument**	synapt		
**Date**	15-Sep-19		

Chromatogram

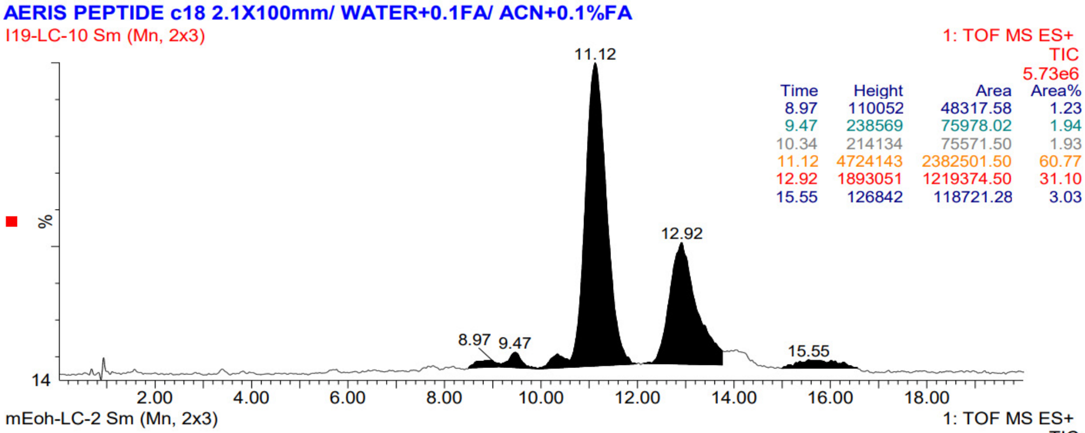

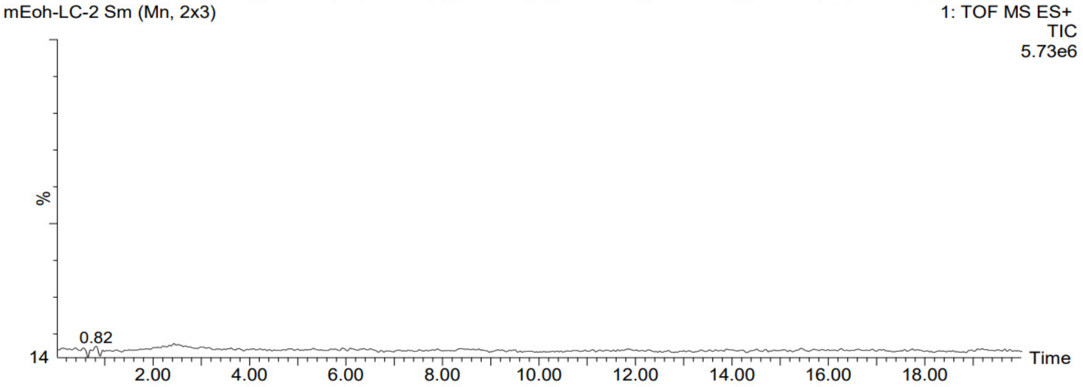

MS spectrum

AERIS PEPTIDE c18 2.1X100mm/ WATER+0.1FA/ ACN+0.1%FA

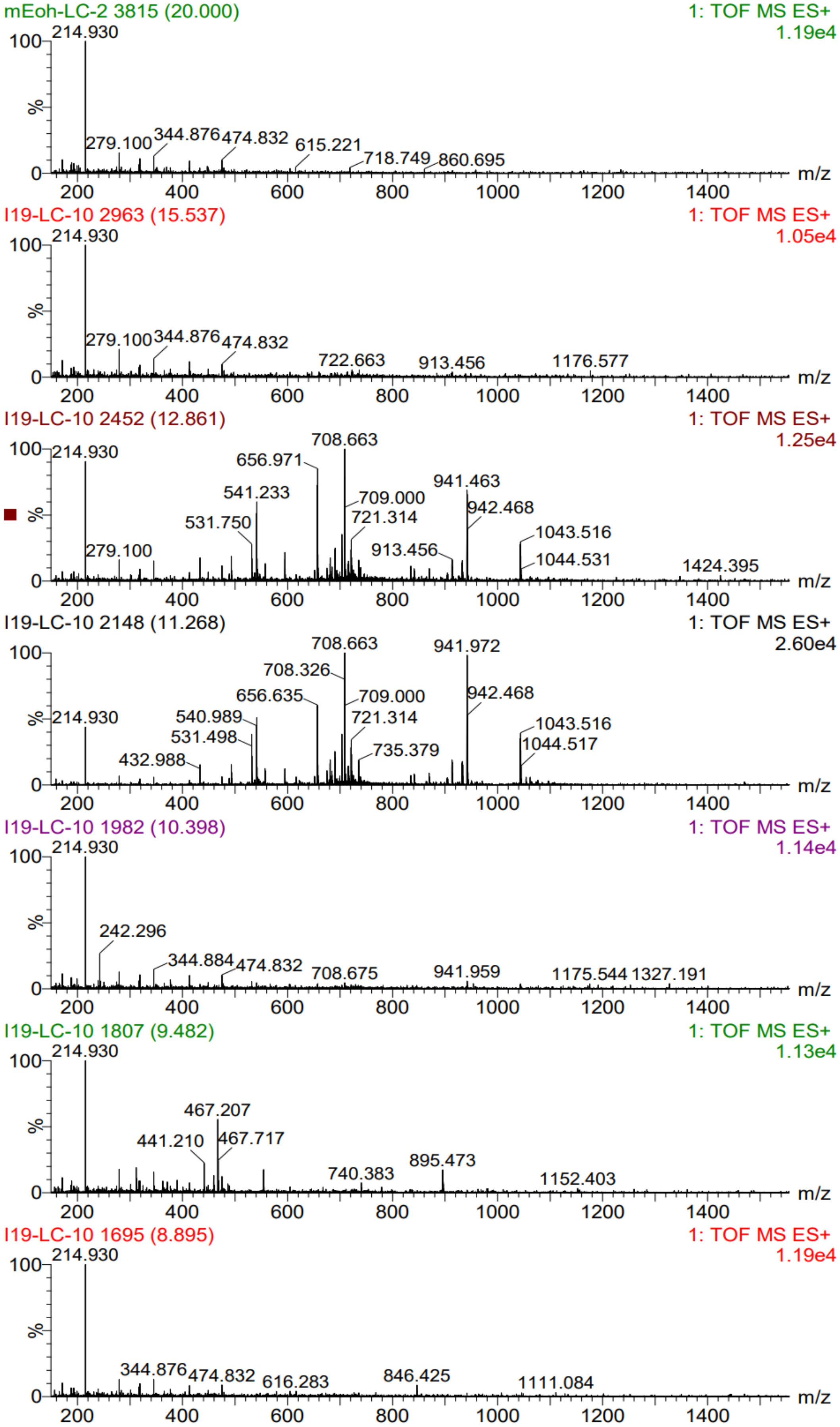

Preliminary perifusion tests of human islets

Figure 1S displays very much similar line shapes of human islets in response to stimuli despite varying degrees of insulin released. Based off these initial observations, we focused on quantifying the amount of insulin released during the primary response as a gauge for evaluation. This figure has been added in the supporting information.

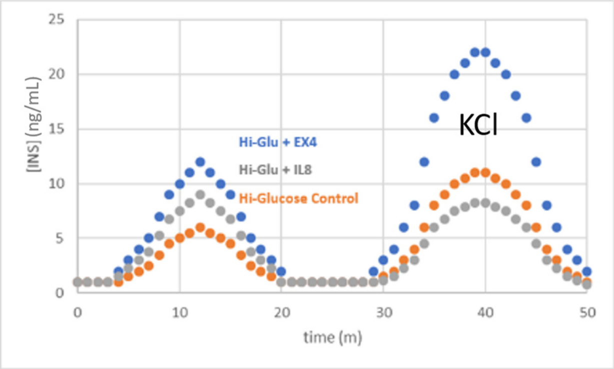

Figure S1: Dynamic stimulation of human islets in response to stimuli. Primary stimulation (5-20m) is followed by membrane depolarization by KCl (30-50m).

Gene Expression

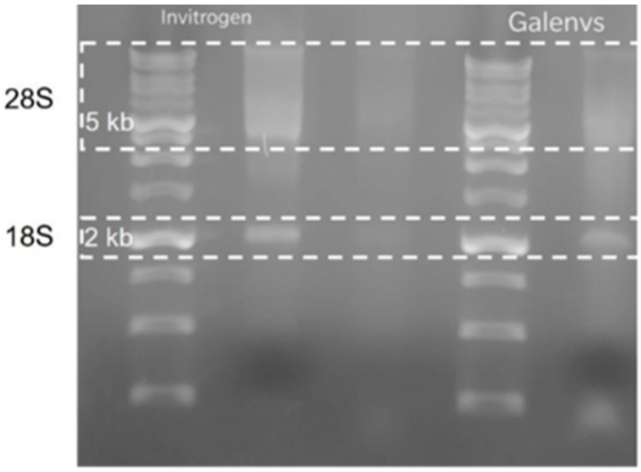

Figure S2 - Gel electrophoresis of purified RNA using Invitrogen kit

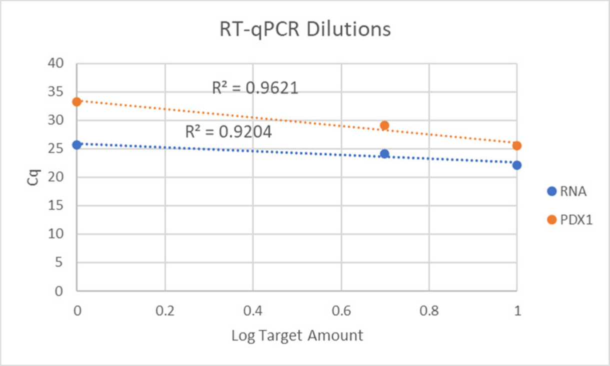

Figure S3 - Linear dependence of threshold amplification validates qPCR primers and RNA extraction
